# A Report of the Trees, Shrubs, and Plants, Used by the Indians of Upper Canada as Medicine, and for the Purposes of Dyeing, &c.; with Their Indian Names

**Published:** 1824-03

**Authors:** Robert Kerr

**Affiliations:** senior Surgeon to the Indian Department in both the Canadas.


Art. VII.—*A Report of the Trees, Shrubs, and Plants, used by the Indians of Upper Canada as Medicine, and for the Purposes of Dyeing, &c.; with their Indian Names.* By Robert Kerr, Esq. senior Surgeon to the Indian Department in both the Canadas.

Acorus Calamus, *Sweet Flag.*—Indian name, *Ononóron.*

The calamus grows in most parts of Upper Canada, in moist situations; not used as an article of commerce; can be procured in great quantities. The root, when dried, the Indians scrape or grate, and give it to children for pains in the stomach and bowels from flatulence; they give it to adults for all complaints in the stomach. They chew the root, and swallow the juice. The best time to dig the root is in the fall of the year; wash it, dry it in the shade.

Aralia Spinora, *Prickly Ash.*—Indian name, *Ojonquanawea.*

The bark and root of this plant are used by the Indians in rheumatism and syphilis. It promotes perspiration. It is one of the ingredients in the famous Indian decoction used in the last-mentioned disease. When I come to the article Sarsaparilla, I will give the various roots and barks used by the Indians for that decoction.

It grows in most parts of the province, especially the western parts, and can be procured in great quantities; is not an article of commerce.

Pinus Canadensis, *the Hemlock Tree.*—Indian name, *Onénta*

The Indians use a decoction of the branches in the rheumatism and in colds; and the inner bark of the root they dry and pulverise, and use the powder in venereal ulcers; using at the same time a quart or three pints, in the twenty-four hours, of the Indian decoction, until the cure is effected, which is generally in three weeks or a month. It is astonishing what cures I have seen made in the syphilis, without the smallest use of mercury. I have seen the persons so cured ten years afterwards, and no secondary complaints had then made their appearance; and those persons enjoyed good health.

Not an article of commerce.

Foracinus Juglandi Folia, *the Swamp, or Walnut-leaved Ash.*—Indian name, *Egh-saat.*

The bark of the root the Indians use in decoction, in rheumatism, and, with other ingredients, in syphilis; it promotes perspiration, and is a good diuretic. It is a very common tree in Upper Canada, grows to a great size, and delights in a moist, rich soil. The best time to peel the bark is in the month of July. Not an article of commerce.

Agrimonia Eupatoria, *Agrimony.*—Indian name, *Itendknawthis.*

The root and leaves are used. This plant is a native of Upper Canada. The Indians use an infusion of the root in inflammatory fevers; the leaves are used in the same way, and given to patients that are convalescent from fever. The Indians say it strengthens the stomach.

Arctium Lappa, *Burdock.*—Indian name, *Orhodeiowa.*

The Indians use a decoction of the leaves in the rheumatism, and they dress blisters with the fresh leaves. The leaves are good to apply to the backs of those soldiers that have been severely punished. It grows in great plenty over the province. Not an article of commerce.

Pinus Balsamea, *Canadian Balsam.*—Indian name, *Otshogóton.*

This is a very common tree in both the provinces of Upper and Lower Canada. This tree never grows to a very large size. There arises blisters on the stem of the tree, which the Indians cut open in the winter, and collect the balsam. It has been said that the balsam is collected from the root of the tree, which is no such thing. The Indians use the balsam to fresh wounds: it seems to have the same virtues as the balsam copaiba. I have used it for the same purposes that the balsam copaiba is commonly used, and with success: the dose is generally from twenty-five to forty-five drops. It is exported in small quantities; can be procured in great quantities, and might be made an article of commerce.

No. 301.

Aristolochia Serpentaria, *Snake-root.*—Indian name, *Joderése.*

This plant is found in the western parts of the provence in great quantities, and used by the Indians in intermittents, both in powder and decoction. When intermittents are prevalent in the province, the old settlers use a tincture of the sweet flag and snake-root, infused in whiskey, of which they take a tolerable good allowance in the morning, fasting, by the way of morning-dram. Can be procured in great quantities, and be made an article of commerce, which it is not at present. The best time to dig the root is the fall of the year, and until the frost sets in.

Arum Triphyllum, *Indian Turnip.*—Indian name, *Oqnaróta.*

This plant grows in shady places and swamps; is very hardy. The root is the part used, and is bulbous, resembling in shape a small turnip. In a fresh state it is very acrid, but, when dried perfectly harmless. It is used by the Indians in rheumatism, and in the aphthous sore-throat, in powder mixed with honey.

Cornus Florida, *Dogwood.*—Indian name, *Erharaonahik.*

The bark and the bark of the root is used by the Indians as medicine. This is a very beautiful flowering shrub or tree, with a white flower. The bark is very astringent, (a good substitute for the cinchona.) It may be used in tincture and decoction, as well as in substance; only add one fifth more than when the Peruvian bark is used. I think it possesses both tonic and astringent powers. Might be made an article of commerce, which it is not at present. It might be used to tan leather, with great advantage.

Datura Stramonium, *Thorn-apple.*—Indian name, *Ohickta.*

The leaves and seeds are both used. The Indians use the fresh leaves bruised, when afflicted with rheumatism, with decided effect. I was induced to try the fresh bruised leaves in the gout, and found benefit from the application. The part affected with the gout must be covered with the fresh leaves bruised, and the leaves removed every hour, and fresh leaves applied; giving at the same time small doses of the powdered leaves, say one grain, every twenty-four hours, or half a grain of powdered seeds of the datura. Both the powder of the seeds and leaves to be used with great caution: if used in too great doses, will bring on the most distressing complaints. I think the powder of the leaves the most proper to use. I have applied the bruised leaves to the indurated and inflamed breasts of nursing women, with decided effect. The bruised leaves are an excellent application in the hernia humoralis, or a poultice of the leaves, with a little sweet oil. The best time to gather the leaves is when the plant is in full bloom; the seeds to be gathered when ripe. Both the powdered leaves and seeds to be kept in close-stopped phials.

JUGLANS CINEREA, *Butter-nut.*—Indian name, *Okyewáta.*

The inner bark is the part used for medicinal purpose.

During the first American war, owing to the vessel coming from Europe with the medicines for the army in Canada being captured by the enemy, we were in want of medicines, especially at the garrisons in that part of Canada now called Upper Canada. I was forced, from necessity, to make an extract of the juglans cinerea, and found it to answer almost as well as jalap; from fifteen to thirty grains a dose. Joined with the submurias hydrarg. an excellent purge in bilious fevers. In small doses, it is good in dysentery and in habitual costiveness. The extract of the juglans cinerea is easily prepared, in the same manner as the extract of the bark is prepared, and to be close covered. The juglans cinerea grows in most parts of Upper Canada; the wood is called *white walnut.* Both wood and bark might be made an article of commerce, but it is not so at present. Grows all over the province.

LAURUS SASSAFRAS, *Sassafras.*—Indian name, *Atstaas.*

The bark, the bark of the root, and the root, are used by the Indians for medicinal purposes. It is to be found in most parts of Upper Canada. The Indians use the decoction in colds and in syphilis. It has been exported in small quantities, but never to any great extent. Can be procured in great abundance.

MARUBIUM VULGARE, *Horehound.*—Indian name, *Kaderakerase.*

The whole plant, except the root, is used for medicinal purposes, and grows spontaneously in many parts of Upper Canada. Its virtues seem to be stronger than in Europe. The Indians use it as a pectoral in colds, and as a tonic and astringent in intermittents. From necessity, I have used it in both complaints, and with success. Not an article of commerce.

PODOPHYLLUM PELATUM, *May Apple.*—Indian name, *Oneahotsdé.*

The root is the part used for medicinal purposes. The May apple is common in every part of Upper Canada, growing spontaneously in low shady situations. It is a sure purgative; one scruple is a dose for an adult, the root being reduced to a fine powder; and may be joined with the submurias hydrarg. with great advantage.

The Indians have a method of roasting the roots in the hot ashes of a wood fire, until they are of a mealy whiteness in the inside; then break them to pieces, and boil them in six gills of soft water to one gill: given to the patient at bed-time, it operates early in the next morning: Six roots are the number to be so used for an adult. The Indians use the May-apple for all eruptions of the skin, and a foulness of the blood. The roots ought to be gathered in the fall of the year, when the leaf begins to turn yellow.

PYROLA UMBELLATA, *Winter Green.*—Indian name, *Onounquaat.*

The whole of this plant is used for medicinal purposes; it is found in every part of Upper and Lower Canada. The Indians use it in dropsy and in gonorrhœa. I have used it in both cases, in decoction and infusion; a strong decoction is the best manner of using it. It is a powerful diuretic, and promotes perspiration.

SANGUINERIA CANADENSIS, *Blood-root.*—Indian name, *Thanckwas.*

The root is the part used, and is a common plant, as its name denotes. It is used by the Indians as a paint, and as an emetic, but it must be used with caution. Eight grains of the fresh powdered root is a dose for an adult; when powdered, ought to be kept in close-stopped vessels. The root ought to be taken up in the month of October.

SCUTELLARIA LATERIFLORA, *Blue Scullcap.*—Indian name, *Thanap.*

The whole of this plant is used. It is very common in Upper Canada; grows on the banks of rivers; flowers in August, which is the best time to gather it. It is used with success in canine madness. The Indians make a strong decoction of the plant; take four ounces four times a-day, until the cure is effected; keeping the body open at the same time with the juglans cinerea.

SMILAX SARSAPARILLA, *Sarsaparilla.*—Indian name, *Yeyenthos Ononqua.*

This root is found in the midland and western districts of this province, in great quantities: the Indians use it in the venereal disease, and is the principal ingredient in the famous Indian decoction. The Indians prepare the decoction in the following manner:—They take of the root of sarsaparilla, sliced, one pound; the bark of the root of sassafras, half a pound; the bark of the root of the prickly ash, the same quantity; the bark of the swamp ash, near the root, the same quantity; soft water, two gallons: the barks to be chipped, the root bruised: boil away to one gallon. Half a pint every six hours is given to the patient, or one quart in the twenty-four hours.

Can be procured in great quantities. Not an article of commerce.

PANAX QUINQUIFOLIUM, *Ginseng.*—Indian name, *Oteraagivek.*

The root is the part used. It is a perennial plant, grows in great quantities over most parts of the province; it has an aromatic bitterness. The Indians chew the root, and swallow the juice, to strengthen the stomach after fevers. I have used a tincture of the bruised roots in brandy, to worn-out and debilitated patients, with great success; especially when the cinchona did not agree with them.

RHUS TYPHINUM, *Sumach.*—Indian name, *Tcyeyesta.*

It grows very plentifully over Upper Canada. The tree rises to the height of ten or twelve feet; the young branches are covered with a soft velvet down; the flowers are produced in close tufts at the end of the branches, and are succeeded by seeds enclosed in purple woolly covers: this part, with the leaves, they use in dyeing, and give a deep permanent black; and I am told may be used in tanning, instead of oak-bark, and answers much better for tanning fine skins for gloves than any other substance we yet know of. Can be procured in great quantities, and, if properly known, might be made an article of commerce; at present not exported.

ULMUS ASPERA, *Red or Slipping Elm.*—Indian name, *Ohoktsera.*

The inner bark is the part used. The Indians use a poultice of the inner bark for gun-shot wounds and for burns, with great effect; likewise for ill-conditioned ulcers. I have often used the ulmus aspera for burns, with great success. A decoction of the inner bark is used by the Indians in inflammatory fevers. Can be procured in great quantities; not an article of commerce.

ZANTHORHISA SIMPLICISSIMA, *Yellow Root.*—Indian name, *Yovdaweasirònde.*

The root is the part used: it is a strong and pleasant bitter, and will sit easy on the stomach in doses of twenty to thirty grains of the powdered root. I think it might make a good substitute for the columba.

It has been used as a dye, and imparts a drab colour to woollen cloths, and a very fine yellow colour to silk; but the dye will not take on cotton or linen. It grows in moist marshy ground, and in a rich soil. Not an article of commerce.

*Fort George, Upper Canada; September* 18*th*, 1823.

